# Crystal structures of (2,2′-bipyridyl-κ^2^ *N*,*N*′)bis­[*N*,*N*-bis­(2-hydroxy­eth­yl)di­thio­carbamato-κ^2^ *S*,*S*′]zinc dihydrate and (2,2′-bipyridyl-κ^2^ *N*,*N*′)bis­[*N*-(2-hydroxy­eth­yl)-*N*-iso­propyl­dithio­carbamato-κ^2^ *S*,*S*′]zinc

**DOI:** 10.1107/S2056989016000700

**Published:** 2016-01-20

**Authors:** Siti Artikah M. Safbri, Siti Nadiah Abdul Halim, Edward R. T. Tiekink

**Affiliations:** a Department of Chemistry, University of Malaya, 50603 Kuala Lumpur, Malaysia; b Centre for Crystalline Materials, Faculty of Science and Technology, Sunway University, 47500 Bandar Sunway, Selangor Darul Ehsan, Malaysia

**Keywords:** crystal structure, zinc, di­thio­carbamate, hydrogen bonding

## Abstract

The common feature of the title compounds, [Zn(C_5_H_10_NO_2_S_2_)_2_(C_10_H_8_N_2_)]·2H_2_O, (I), and [Zn(C_6_H_12_NOS_2_)_2_(C_10_H_8_N_2_)], (II), is the location of the Zn^II^ atoms on a twofold rotation axis. Further, each Zn^II^ atom is chelated by two symmetry-equivalent and symmetrically coordinating di­thio­carbamate ligands and a 2,2′-bi­pyridine ligand. The resulting N_2_S_4_ coordination geometry is based on a highly distorted octa­hedron in each case. In the mol­ecular packing of (I), supra­molecular ladders mediated by O—H⋯O hydrogen bonding are found whereby the uprights are defined by {⋯HO(water)⋯HO(hy­droxy)⋯}_*n*_ chains parallel to the *a* axis and with the rungs defined by ‘Zn[S_2_CN(CH_2_CH_2_)_2_]_2_’. The water mol­ecules connect the ladders into a supra­molecular layer parallel to the *ab* plane *via* water-O—H⋯S and pyridyl-C—H⋯O(water) inter­actions, with the connections between layers being of the type pyridyl-C—H⋯S. In (II), supra­molecular layers parallel to the *ab* plane are sustained by hy­droxy-O—H⋯S hydrogen bonds with connections between layers being of the type pyridyl-C—H⋯S.

## Abstract 2

The Zn^II^ atom in each of [Zn{S_2_CN(CH_2_CH_2_OH)_2_}_2_(bipy)]·2H_2_O, (I), and [Zn{S_2_CN(*i*Pr)CH_2_CH_2_OH}_2_(bipy)], (II), is coordinated symmetrically by two di­thio­carbamate ligands and a 2,2′-bi­pyridine ligand resulting in an N_2_S_4_ donor set that defines a heavily distorted octa­hedral geometry. The mol­ecular packing features significant hydrogen bonding in each case with supra­molecular ladders found in (I) sustained by O—H⋯O hydrogen bonds, and layers in (II) sustained by O—H⋯S hydrogen bonds.

## Chemical context   

The di­thio­carbamate ligand ^−^S_2_CN*RR*′, is well known as an effective chelator of transition metals, main group elements and lanthanides (Hogarth, 2005 ▸; Heard, 2005 ▸). The resulting four-membered *M*S_2_C chelate ring has metalloaromatic character (Masui, 2001 ▸) and may act as an acceptor for C—H⋯π(chelate) inter­actions (Tiekink & Zukerman-Schpector, 2011 ▸) much in the same way as the now widely accepted C—H⋯π(arene) inter­actions. While other 1,1-di­thiol­ate species may also form analogous inter­actions – these were probably first discussed in cadmium xanthate (^−^S_2_CO*R*) structures (Chen *et al.*, 2003 ▸) – di­thio­carbamate compounds have a greater propensity to form C—H⋯π(chelate) inter­actions, an observation related to the relatively greater contribution of the canonical structure ^2−^S_2_C=N^+^ *RR*′ to the overall electronic structure that enhances the electron density in the chelate ring (Tiekink & Zukerman-Schpector, 2011 ▸). This factor explains the strong chelation ability of the di­thio­carbamate ligand and at the same time accounts for the reduced Lewis acidity of the metal cation in metal di­thio­carbamates which reduces the ability of these species to form extended architectures in their inter­actions with Lewis bases. One way of overcoming the relative inability of the metal cation to engage in supra­molecular association is to function­alize the di­thio­carbamate ligand with, relevant to the present report, hydrogen-bonding functionality. In this context and as a continuation of earlier studies of the zinc-triad elements with di­thio­carbamate ligands featuring hy­droxy­ethyl groups capable of forming hydrogen-bonding inter­actions (Benson *et al.*, 2007 ▸; Broker & Tiekink, 2011 ▸; Zhong *et al.*, 2004 ▸; Tan *et al.*, 2013 ▸, 2016 ▸; Safbri *et al.*, 2016 ▸; Howie *et al.*, 2009 ▸), herein, the crystal and mol­ecular structures of two new zinc di­thio­carbamates, Zn[S_2_CN(CH_2_CH_2_OH)_2_]_2_(bipy)·2H_2_O, (I), and Zn[S_2_CN(*i*Pr)CH_2_CH_2_OH]_2_(bipy), (II) where bipy = 2,2′-bi­pyridine are described.
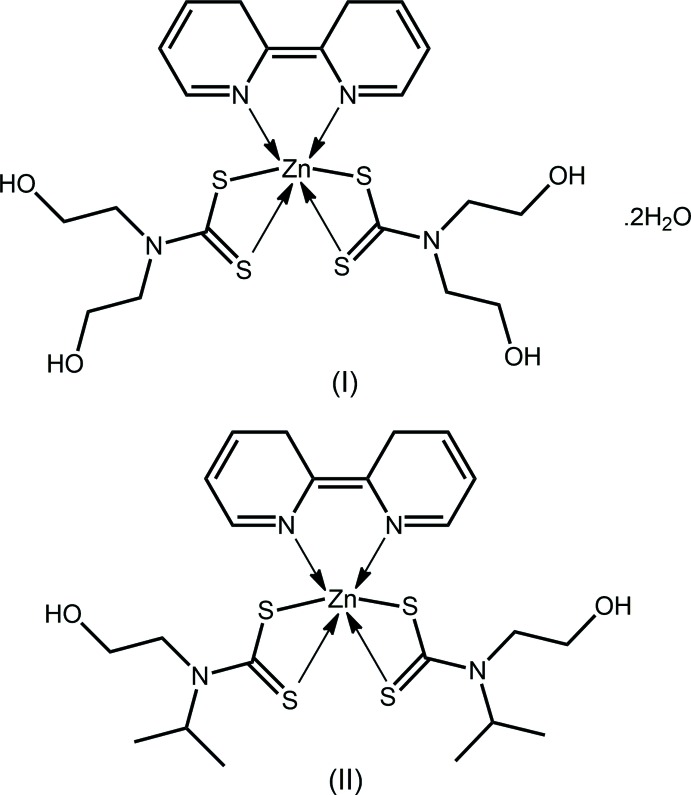


## Structural commentary   

**Figure 1:**
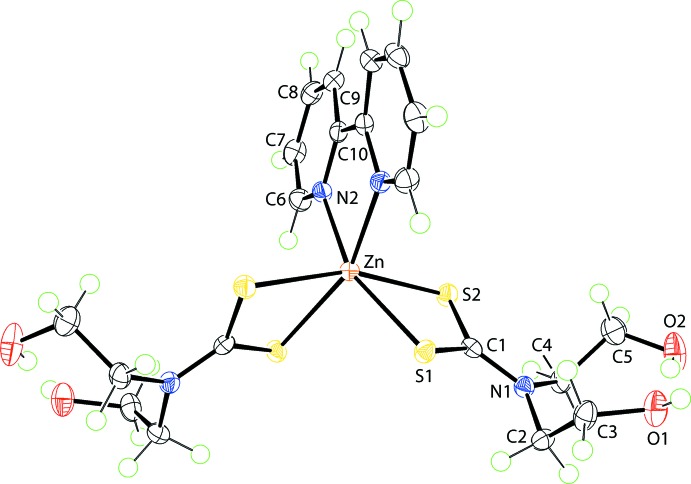
The mol­ecular structure of the zinc compound in (I), showing the atom-labelling scheme and displacement ellipsoids at the 70% probability level; the water mol­ecules of crystallization have been omitted. The unlabelled atoms are related by the symmetry operation 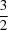 − *x*, 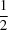 − *y*, *z*.

The mol­ecular structure of the zinc compound in (I) is shown in Fig. 1 ▸ and selected geometric parameters are given in Table 1 ▸. The zinc cation is located on a twofold rotation axis and is chelated by two symmetry-equivalent di­thio­carbamate ligands and the 2,2′-bi­pyridine ligand, which is bis­ected by the twofold rotation axis. The di­thio­carbamate ligand chelates in a symmetric mode with the difference between the Zn—S_long_ and Zn—S_short_ bond lengths being 0.02 Å. The shorter Zn—S bond is approximately *trans* to a pyridyl-N atom. The N_2_S_4_ coordination geometry is based on an octa­hedron. In this description, one triangular face is defined by the S1, S2^i^ and N2^i^ atoms, and the other by the symmetry equivalent atoms [symmetry code: (i) 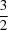 − *x*, 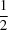 − *y*, *z*]. The dihedral angle between the two faces is 3.07 (4)° and the twist angle between them is approximately 35°, *cf*. 0 and 60° for ideal trigonal–prismatic and octa­hedral angles, respectively. The twist toward a trigonal prism is related in part to the acute bite angles subtended by the chelating ligands (Table 1 ▸).

Compound (I) was characterized herein as a dihydrate and may be compared with an unsolvated literature precedent (Deng *et al.*, 2007 ▸) for which selected geometric data are also collected in Table 1 ▸. First and foremost, the mol­ecular symmetry observed in unsolvated (I) is lacking. Also, the range of Zn—S bond lengths is significantly broader at 0.14 Å, but the trend that the shorter Zn—S bonds are approximately *trans* to the pyridyl-N atoms persists. The dihedral angle between the trigonal faces is 5.33 (6)° and the twist between them is 31°, indicating an inter­mediate coordination geometry.

**Figure 2:**
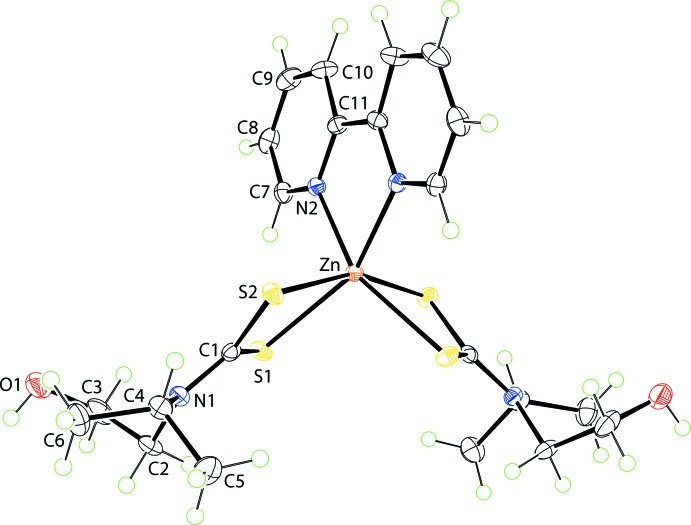
The mol­ecular structure of (II), showing the atom-labelling scheme and displacement ellipsoids at the 70% probability level. The unlabelled atoms are related by the symmetry operation 1 − *x*, *y*, 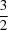 − *z*.

The mol­ecule of compound (II) (Fig. 2 ▸) is also located about a twofold rotation axis and presents geometric features closely resembling those of (I), Table 1 ▸. The angle between the triangular faces is 1.50 (5)° and the twist angle is approximately 30°, again indicating a highly distorted coordination geometry.

## Supra­molecular features   

Geometric parameters characterizing the inter­molecular inter­actions operating in the crystal structures of (I) and (II) are collected in Tables 2 ▸ and 3 ▸, respectively.

**Figure 3:**
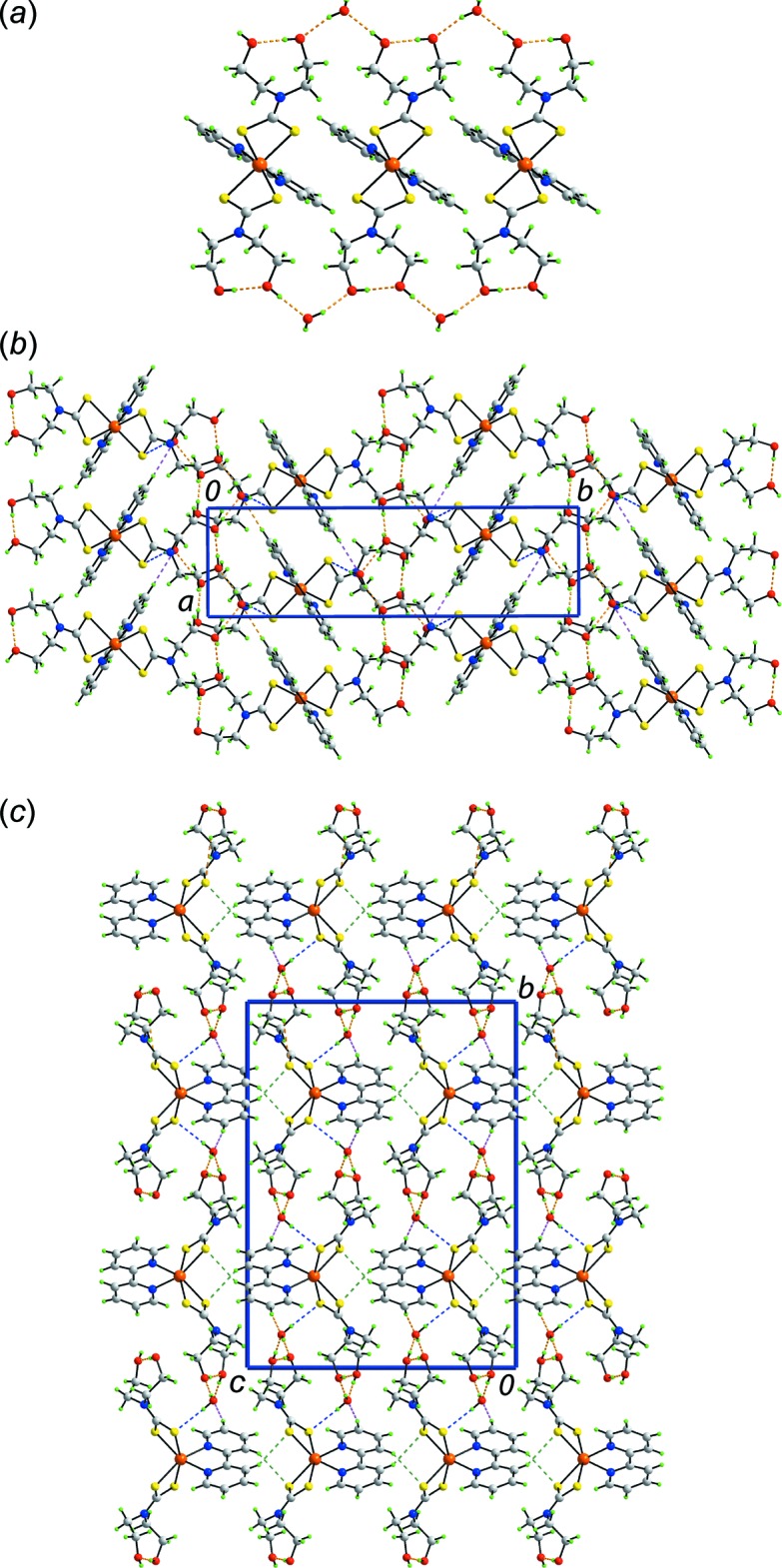
Mol­ecular packing in (I), showing (*a*) the supra­molecular ladders aligned along the *a* axis and sustained by O—H⋯O hydrogen bonding, (*b*) the supra­molecular layers parallel to the *ab* plane whereby the ladders in (*a*) are connected by O—H⋯S and C—H⋯O inter­actions, and (*c*) a view of the unit-cell contents in projection down the *a* axis, showing C—H⋯S inter­actions along the *c* axis connecting the layers in (*b*). The O—H⋯O, O—H⋯S, C—H⋯O and C—H⋯S inter­actions are shown as orange, blue, pink and green dashed lines, respectively.

In the mol­ecular packing of (I), supra­molecular ladders mediated by O—H⋯O hydrogen bonding are found. There is an intra­molecular hy­droxy-O—H⋯O(hy­droxy) hydrogen bond as well as inter­molecular hy­droxy-O—H⋯O(water) and water-O—H⋯O(hy­droxy) hydrogen bonds. This mode of association results in supra­molecular {⋯HO(water)⋯HO(hy­droxy)⋯HO(hy­droxy)⋯}_*n*_ jagged chains parallel to the *a* axis that serve as the uprights in the supra­molecular ladders whereby the rungs are defined by ‘Zn(S_2_CN(CH_2_CH_2_)_2_’ (Fig. 3 ▸ *a*). The water mol­ecules are pivotal in connecting the ladders into a supra­molecular layer parallel to the *ab* plane by forming water-O—H⋯S and pyridyl-C—H⋯O(water) inter­actions (Fig. 3 ▸ *b*). The connections between layers to consolidate the three-dimensional architecture are of the type pyridyl-C—H⋯S (Fig. 3 ▸ *c*).

Naturally, the mol­ecular packing in the unsolvated form of (I) is distinct (Deng *et al.*, 2007 ▸). However, a detailed analysis of the packing is restricted as one of the hy­droxy groups is disordered over two sites. Further, there are large voids in the crystal structure, amounting to approximately 570 Å^3^ or 19.2% of the available volume (Spek, 2009 ▸). This is reflected in the crystal packing index of 59.2% which compares to 71.3% in (I). Globally, the crystal structure comprises alternating layers of hydro­philic and hydro­phobic regions with the former arranged as supra­molecular rods, indicating significant hydrogen bonding in this region of the crystal structure.

**Figure 4:**
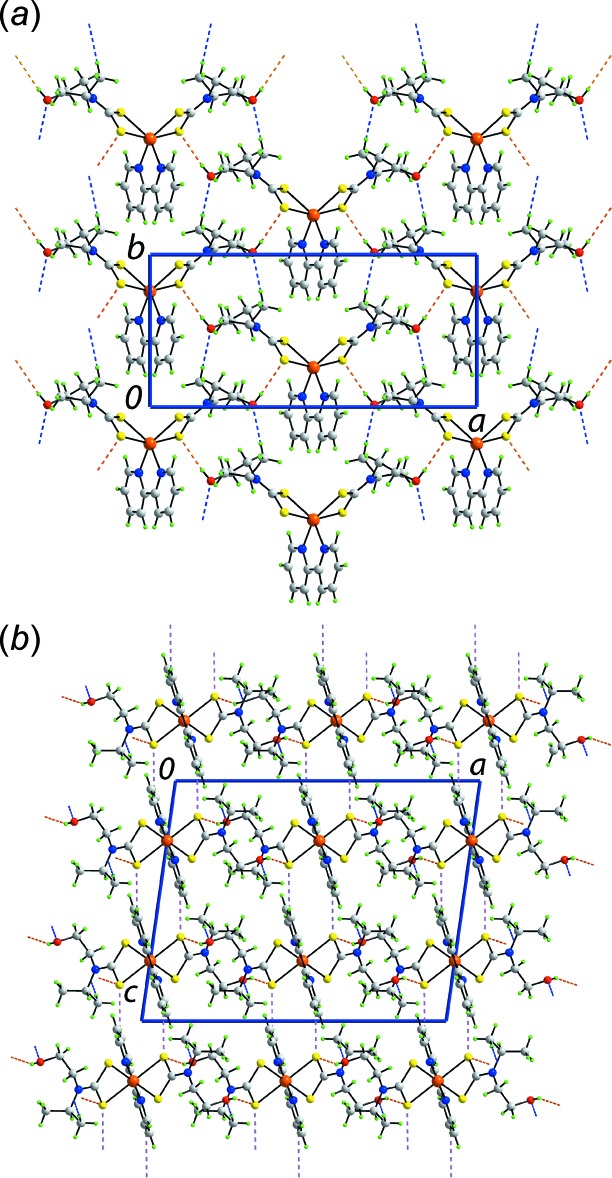
Mol­ecular packing in (II), showing (*a*) the supra­molecular layers parallel to the *ab* plane sustained by O—H⋯S and C—H⋯O inter­actions, and (*b*) a view of the unit-cell contents in projection down the *b* axis, showing C—H⋯S inter­actions along the *c* axis connecting the layers in (*b*). The O—H⋯S, C—H⋯O and C—H⋯S inter­actions are shown as orange, blue and pink dashed lines, respectively.

In the mol­ecular packing of (II), hy­droxy-O—H⋯S hydrogen bonds lead to supra­molecular layers parallel to the *ab* plane (Fig. 4 ▸ *a*). Additional stabilization to this arrangement is provided by methyl-C—H⋯O(hy­droxy) inter­actions. Connections between layers to consolidate the three-dimensional packing are of the type pyridyl-C—H⋯S (Fig. 4 ▸ *b*).

## Database survey   

Binary zinc di­thio­carbamates are generally binuclear as a result of the presence of chelating and tridentate, μ_2_-bridging ligands, leading to penta-coordinate geometries (Tiekink, 2003 ▸). The exceptional structures arise when the steric bulk of at least one of the terminal substituents is too great to allow for supra­molecular association, *e.g. R* = cyclo­hexyl (Cox & Tiekink, 2009 ▸) and *R* = benzyl (Decken *et al.*, 2004 ▸). However, there is a subtle energetic balance between the two forms as seen in the crystal structure of Zn[S_2_CN(*i*-Bu)_2_]_2_ which comprises equal numbers of mono- and bi-nuclear mol­ecules (Ivanov *et al.*, 2005 ▸). As the *R* groups are generally aliphatic, there is limited scope for controlled supra­molecular aggregation between the mol­ecules. This changes in the case of the present study as at least one *R* group has an hy­droxy­ethyl substituent. Indeed, a rich tapestry of structures have been observed for zinc compounds with this family of di­thio­carbamate ligands.

The common feature of the mol­ecular structures of the known binary species, Zn[S_2_NC(*R*)CH_2_CH_2_OH]_2_, is the adoption of a binuclear motif (Benson *et al.*, 2007 ▸; Tan *et al.*, 2015 ▸). In the mol­ecular packing of these species, when *R* = CH_2_CH_2_OH, a three-dimensional architecture is constructed based on hydrogen bonding (Benson *et al.*, 2007 ▸). When the hydrogen-bonding potential is reduced, as in the case when *R* = Et, linear supra­molecular chains are formed (Benson *et al.*, 2007 ▸). When *R* = Me, and in the 2:1 adduct with the bridging ligand (3-pyrid­yl)CH_2_N(H)C(=O)C(=O)N(H)CH_2_(3-pyrid­yl), inter­woven supra­molecular chains are formed based on hydrogen bonding (Poplaukhin & Tiekink, 2010 ▸). Extensive hydrogen bonding is also noted in co-crystals, *e.g*. for *R* = Me in the 2:1 adduct with (3-pyrid­yl)CH_2_N(H)C(=S)C(=S)N(H)CH_2_(3-pyrid­yl), a 2:1 co-crystal with S_8_ has been characterized in which a two-dimensional array sustained by O—H⋯O hydrogen bonding is found (Poplaukhin *et al.*, 2012 ▸). From the foregoing, it is clear that a rich structural chemistry exists for these compounds, well worthy of further investigation. Complementing these inter­ests are the observations that zinc compounds with these ligands (Tan *et al.*, 2015 ▸), along with gold (Jamaludin *et al.*, 2013 ▸) and bis­muth (Ishak *et al.*, 2014 ▸) exhibit exciting anti-cancer potential.

## Synthesis and crystallization   

The potassium salts of the di­thio­carbamate anions (Howie *et al.*, 2008 ▸; Tan *et al.*, 2013 ▸) and zinc compounds (Benson *et al.*, 2007 ▸) were prepared in accord with the literature methods. The 1:1 adducts with 2,2′-bi­pyridine were prepared in the following manner. Zn[S_2_CN(CH_2_CH_2_OH)_2_]_2_ (0.20 g, 0.47 mmol) and 2,2′-bi­pyridine (Sigma Aldrich; 0.07 g, 0.47 mmol) were dissolved in acetone (30 ml) and ethanol (10 ml), respectively. The solution of 2,2′-bi­pyridine was added dropwise into the other solution with stirring for about 30 mins, resulting in a change from a colourless to a light-yellow solution. The mixture was left to stand to allow for crystallization and crystals of (I) for X-ray analysis were harvested directly. Compound (II) was prepared and harvested similarly from the reaction of Zn[S_2_CN(*i*Pr)CH_2_CH_2_OH]_2_ (0.20 g, 0.47 mmol) in chloro­form (30 ml) and 2,2′-bi­pyridine (0.07 g, 0.47 mmol) in acetone (10 ml).

## Refinement   

Crystal data, data collection and structure refinement details are summarized in Table 4 ▸. For each of (I) and (II), carbon-bound H atoms were placed in calculated positions (C—H = 0.95–1.00 Å) and were included in the refinement in the riding-model approximation, with *U*_iso_(H) set to 1.2–1.5*U*_eq_(C). The O-bound H atoms were located in a difference Fourier map but were refined with a distance restraint of O—H = 0.84±0.01 Å, and with *U*_iso_(H) set to 1.5*U*_eq_(O).

## Supplementary Material

Crystal structure: contains datablock(s) I, II, global. DOI: 10.1107/S2056989016000700/wm5262sup1.cif

Structure factors: contains datablock(s) I. DOI: 10.1107/S2056989016000700/wm5262Isup2.hkl

Structure factors: contains datablock(s) II. DOI: 10.1107/S2056989016000700/wm5262IIsup3.hkl

CCDC references: 1447175, 1447174

Additional supporting information:  crystallographic information; 3D view; checkCIF report

## Figures and Tables

**Table 1:** Geometric data (Å, °) for (I), unsolvated (I) and for (II)

Parameter	(I)^*a*^	unsolvated (I)	(II)^*b*^
Zn—S1	2.5361 (5)	2.4632 (12)	2.5068 (5)
Zn—S2	2.5163 (5)	2.5968 (13)	2.5247 (5)
Zn—S3	2.5361 (5)	2.5030 (12)	2.5068 (5)
Zn—S4	2.5163 (5)	2.6045 (13)	2.5247 (5)
Zn—N2	2.1682 (15)	2.157 (4)	2.1695 (15)
Zn—N3	2.1682 (15)	2.154 (3)	2.1695 (15)
C—S	1.7198 (18)–1.7253 (18)	1.696 (4)–1.726 (5)	1.7221 (19)–1.7301 (18)
S1—Zn—S2	71.376 (15)	70.46 (4)	71.289 (16)
S3—Zn—S4	71.376 (15)	70.15 (4)	71.289 (16)
N2—Zn—N2	75.71 (8)	74.72 (12)	75.08 (8)

Notes: (*a*) S3, S4 and N3 are S1^i^, S2^i^ and N2^i^ for (i) 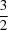 − *x*, 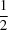 − *y*, *z*; (*b*) S3, S4 and N3 are S1^i^, S2^i^ and N2^i^ for (i) 1 − *x*, *y*, 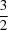 − *z*.

**Table 2:** Hydrogen-bond geometry (Å, °) for (I)

*D*—H⋯*A*	*D*—H	H⋯*A*	*D*⋯*A*	*D*—H⋯*A*
O2—H2*O*⋯O1	0.83 (2)	1.87 (2)	2.696 (2)	177 (3)
O1—H1*O*⋯O1*W*	0.83 (2)	1.88 (2)	2.7115 (19)	177 (2)
O1*W*—H1*W*⋯O2^i^	0.83 (2)	1.91 (2)	2.7216 (19)	166 (2)
O1*W*—H2*W*⋯S2^ii^	0.83 (2)	2.45 (2)	3.2733 (15)	170 (2)
C7—H7⋯O1*W* ^iii^	0.95	2.58	3.517 (2)	171
C6—H6⋯S2^iv^	0.95	2.81	3.490 (2)	129
C9—H9⋯S1^v^	0.95	2.84	3.6857 (18)	149

Symmetry codes: (i) 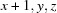; (ii) 
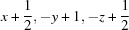
; (iii) 
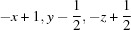
; (iv) 
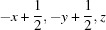
; (v) 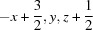.

**Table 3:** Hydrogen-bond geometry (Å, °) for (II)

*D*—H⋯*A*	*D*—H	H⋯*A*	*D*⋯*A*	*D*—H⋯*A*
O1—H1*O*⋯S2^i^	0.84 (2)	2.45 (2)	3.2437 (16)	160 (2)
C5—H5*B*⋯O1^i^	0.98	2.54	3.512 (2)	175
C9—H9⋯S2^ii^	0.95	2.86	3.550 (2)	130

Symmetry codes: (i) 
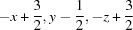
; (ii) 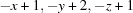.

**Table 4:** Experimental details

	(I)	(II)
Crystal data		
Chemical formula	[Zn(C_5_H_10_NO_2_S_2_)_2_(C_10_H_8_N_2_)]·2H_2_O	[Zn(C_6_H_12_NOS_2_)_2_(C_10_H_8_N_2_)]
*M*_r_	618.10	578.12
Crystal system, space group	Orthorhombic, *P* *c* *c* *n*	Monoclinic, *C*2/*c*
Temperature (K)	100	100
*a*, *b*, *c* (Å)	6.7730 (3), 23.1063 (11), 16.9483 (8)	19.4997 (11), 9.0027 (5), 15.5352 (8)
α, β, γ (°)	90, 90, 90	90, 98.031 (5), 90
*V* (Å^3^)	2652.4 (2)	2700.5 (3)
*Z*	4	4
Radiation type	Mo *K*α	Mo *K*α
μ (mm^−1^)	1.28	1.25
Crystal size (mm)	0.40 × 0.30 × 0.20	0.25 × 0.25 × 0.15

Data collection		
Diffractometer	Agilent SuperNova Dual diffractometer with an Atlas detector	Agilent SuperNova Dual diffractometer with Atlas detector
Absorption correction	Multi-scan (*CrysAlis PRO*; Agilent, 2012 ▸)	Multi-scan (*CrysAlis PRO*; Agilent, 2012 ▸)
*T*_min_, *T*_max_	0.778, 1.000	0.737, 1.000
No. of measured, independent and observed [*I* > 2σ(*I*)] reflections	21039, 3047, 2607	11190, 3095, 2657
*R*_int_	0.049	0.048
(sin θ/λ)_max_ (Å^−1^)	0.650	0.650

Refinement		
*R*[*F* ^2^ > 2σ(*F* ^2^)], *wR*(*F* ^2^), *S*	0.027, 0.066, 1.02	0.030, 0.073, 1.03
No. of reflections	3047	3095
No. of parameters	171	155
No. of restraints	4	1
Δρ_max_, Δρ_min_ (e Å^−3^)	0.39, −0.34	0.38, −0.35

Computer programs: *CrysAlis PRO* (Agilent, 2012 ▸), *SHELXS97* (Sheldrick, 2008 ▸), *SHELXL2014* (Sheldrick, 2015 ▸), *ORTEP-3 for Windows* (Farrugia, 2012 ▸), *DIAMOND* (Brandenburg, 2006 ▸) and *publCIF* (Westrip, 2010 ▸).

## supplementary crystallographic information

### (I) (2,2'-Bipyridyl-κ2N,N')bis[N,N-bis(2-hydroxyethyl)dithiocarbamato-κ2S,S']zinc dihydrate Crystal data

**Table:**

[Zn(C_5_H_10_NO_2_S_2_)_2_(C_10_H_8_N_2_)]·2H_2_O	*D*_x_ = 1.548 Mg m^−^^3^
*M**_r_* = 618.10	Mo *K*α radiation, λ = 0.71073 Å
Orthorhombic, *P**c**c**n*	Cell parameters from 5870 reflections
*a* = 6.7730 (3) Å	θ = 2.6–27.5°
*b* = 23.1063 (11) Å	µ = 1.28 mm^−^^1^
*c* = 16.9483 (8) Å	*T* = 100 K
*V* = 2652.4 (2) Å^3^	Prism, light-yellow
*Z* = 4	0.40 × 0.30 × 0.20 mm
*F*(000) = 1288	

### (I) (2,2'-Bipyridyl-κ2N,N')bis[N,N-bis(2-hydroxyethyl)dithiocarbamato-κ2S,S']zinc dihydrate Data collection

**Table:**

Agilent SuperNova Dual diffractometer with an Atlas detector	3047 independent reflections
Radiation source: SuperNova (Mo) X-ray Source	2607 reflections with *I* > 2σ(*I*)
Mirror monochromator	*R*_int_ = 0.049
Detector resolution: 10.4041 pixels mm^-1^	θ_max_ = 27.5°, θ_min_ = 2.6°
ω scan	*h* = −8→8
Absorption correction: multi-scan (*CrysAlis PRO*; Agilent, 2012)	*k* = −30→29
*T*_min_ = 0.778, *T*_max_ = 1.000	*l* = −22→21
21039 measured reflections	

### (I) (2,2'-Bipyridyl-κ2N,N')bis[N,N-bis(2-hydroxyethyl)dithiocarbamato-κ2S,S']zinc dihydrate Refinement

**Table:**

Refinement on *F*^2^	4 restraints
Least-squares matrix: full	Hydrogen site location: mixed
*R*[*F*^2^ > 2σ(*F*^2^)] = 0.027	*w* = 1/[σ^2^(*F*_o_^2^) + (0.0256*P*)^2^ + 1.8882*P*] where *P* = (*F*_o_^2^ + 2*F*_c_^2^)/3
*wR*(*F*^2^) = 0.066	(Δ/σ)_max_ = 0.001
*S* = 1.02	Δρ_max_ = 0.39 e Å^−^^3^
3047 reflections	Δρ_min_ = −0.34 e Å^−^^3^
171 parameters	

### (I) (2,2'-Bipyridyl-κ2N,N')bis[N,N-bis(2-hydroxyethyl)dithiocarbamato-κ2S,S']zinc dihydrate Special details

**Table:**

Geometry. All esds (except the esd in the dihedral angle between two l.s. planes) are estimated using the full covariance matrix. The cell esds are taken into account individually in the estimation of esds in distances, angles and torsion angles; correlations between esds in cell parameters are only used when they are defined by crystal symmetry. An approximate (isotropic) treatment of cell esds is used for estimating esds involving l.s. planes.

### (I) (2,2'-Bipyridyl-κ2N,N')bis[N,N-bis(2-hydroxyethyl)dithiocarbamato-κ2S,S']zinc dihydrate Fractional atomic coordinates and isotropic or equivalent isotropic displacement parameters (Å^2^)

**Table:**

	*x*	*y*	*z*	*U*_iso_*/*U*_eq_	
Zn	0.7500	0.2500	0.25218 (2)	0.01179 (9)	
S1	0.88162 (6)	0.32477 (2)	0.15608 (3)	0.01323 (11)	
S2	0.48554 (6)	0.32443 (2)	0.22814 (3)	0.01403 (11)	
N1	0.5960 (2)	0.39933 (6)	0.11619 (8)	0.0123 (3)	
N2	0.5960 (2)	0.21427 (6)	0.35319 (8)	0.0122 (3)	
O1	0.8068 (2)	0.51852 (6)	0.09325 (9)	0.0221 (3)	
H1O	0.899 (3)	0.5406 (9)	0.1051 (14)	0.033*	
O2	0.4459 (2)	0.52831 (6)	0.15907 (9)	0.0273 (3)	
H2O	0.559 (2)	0.5263 (11)	0.1401 (14)	0.041*	
O1W	1.1132 (2)	0.58995 (6)	0.12670 (8)	0.0202 (3)	
H1W	1.225 (2)	0.5761 (10)	0.1348 (14)	0.030*	
H2W	1.096 (3)	0.6133 (8)	0.1634 (10)	0.030*	
C1	0.6497 (3)	0.35475 (8)	0.16217 (10)	0.0123 (4)	
C2	0.7307 (3)	0.42078 (8)	0.05455 (10)	0.0151 (4)	
H2A	0.6521	0.4409	0.0137	0.018*	
H2B	0.7958	0.3872	0.0291	0.018*	
C3	0.8882 (3)	0.46162 (8)	0.08437 (11)	0.0178 (4)	
H3A	0.9390	0.4478	0.1358	0.021*	
H3B	0.9996	0.4627	0.0466	0.021*	
C4	0.3983 (3)	0.42555 (8)	0.12367 (11)	0.0151 (4)	
H4A	0.3018	0.3948	0.1369	0.018*	
H4B	0.3597	0.4422	0.0721	0.018*	
C5	0.3875 (3)	0.47266 (8)	0.18629 (12)	0.0200 (4)	
H5A	0.2503	0.4750	0.2061	0.024*	
H5B	0.4731	0.4615	0.2311	0.024*	
C6	0.4312 (3)	0.18264 (8)	0.34890 (11)	0.0161 (4)	
H6	0.3897	0.1689	0.2988	0.019*	
C7	0.3183 (3)	0.16904 (8)	0.41461 (11)	0.0170 (4)	
H7	0.2017	0.1465	0.4096	0.020*	
C8	0.3791 (3)	0.18905 (8)	0.48773 (11)	0.0154 (4)	
H8	0.3034	0.1810	0.5336	0.019*	
C9	0.5519 (3)	0.22098 (8)	0.49310 (10)	0.0140 (4)	
H9	0.5975	0.2346	0.5428	0.017*	
C10	0.6574 (2)	0.23279 (7)	0.42442 (10)	0.0115 (3)	

### (I) (2,2'-Bipyridyl-κ2N,N')bis[N,N-bis(2-hydroxyethyl)dithiocarbamato-κ2S,S']zinc dihydrate Atomic displacement parameters (Å^2^)

**Table:**

	*U*^11^	*U*^22^	*U*^33^	*U*^12^	*U*^13^	*U*^23^
Zn	0.01403 (15)	0.01109 (16)	0.01025 (15)	0.00054 (11)	0.000	0.000
S1	0.0126 (2)	0.0131 (2)	0.0140 (2)	0.00237 (17)	0.00144 (16)	0.00154 (16)
S2	0.0142 (2)	0.0131 (2)	0.0149 (2)	0.00035 (17)	0.00304 (17)	0.00135 (16)
N1	0.0122 (7)	0.0119 (7)	0.0127 (7)	0.0013 (6)	−0.0001 (6)	−0.0003 (6)
N2	0.0126 (7)	0.0115 (7)	0.0124 (7)	0.0007 (6)	−0.0003 (6)	−0.0006 (6)
O1	0.0191 (7)	0.0139 (7)	0.0332 (8)	−0.0013 (6)	0.0019 (6)	0.0008 (6)
O2	0.0186 (7)	0.0141 (7)	0.0491 (10)	0.0024 (6)	0.0051 (7)	−0.0030 (6)
O1W	0.0197 (7)	0.0191 (8)	0.0219 (7)	0.0019 (6)	0.0003 (6)	−0.0048 (6)
C1	0.0142 (9)	0.0118 (9)	0.0110 (8)	−0.0004 (7)	−0.0005 (7)	−0.0028 (6)
C2	0.0182 (9)	0.0148 (9)	0.0123 (9)	0.0008 (7)	0.0018 (7)	0.0027 (7)
C3	0.0151 (9)	0.0147 (10)	0.0236 (10)	0.0024 (7)	0.0032 (7)	0.0034 (8)
C4	0.0130 (9)	0.0145 (9)	0.0176 (9)	0.0033 (7)	−0.0019 (7)	0.0004 (7)
C5	0.0168 (9)	0.0196 (10)	0.0235 (10)	0.0026 (8)	0.0020 (8)	−0.0027 (8)
C6	0.0169 (9)	0.0149 (9)	0.0164 (9)	−0.0014 (7)	−0.0036 (7)	−0.0019 (7)
C7	0.0131 (9)	0.0150 (10)	0.0228 (10)	−0.0033 (7)	−0.0017 (7)	0.0023 (8)
C8	0.0137 (9)	0.0157 (9)	0.0169 (9)	0.0007 (7)	0.0027 (7)	0.0047 (7)
C9	0.0159 (9)	0.0131 (9)	0.0129 (9)	0.0007 (7)	−0.0003 (7)	0.0017 (7)
C10	0.0115 (8)	0.0093 (8)	0.0136 (9)	0.0004 (7)	−0.0017 (7)	0.0001 (7)

### (I) (2,2'-Bipyridyl-κ2N,N')bis[N,N-bis(2-hydroxyethyl)dithiocarbamato-κ2S,S']zinc dihydrate Geometric parameters (Å, º)

**Table:**

Zn—N2^i^	2.1682 (15)	C2—C3	1.511 (3)
Zn—N2	2.1682 (14)	C2—H2A	0.9900
Zn—S2^i^	2.5163 (5)	C2—H2B	0.9900
Zn—S2	2.5163 (5)	C3—H3A	0.9900
Zn—S1^i^	2.5361 (5)	C3—H3B	0.9900
Zn—S1	2.5361 (5)	C4—C5	1.522 (3)
S1—C1	1.7198 (18)	C4—H4A	0.9900
S2—C1	1.7253 (18)	C4—H4B	0.9900
N1—C1	1.342 (2)	C5—H5A	0.9900
N1—C2	1.473 (2)	C5—H5B	0.9900
N1—C4	1.475 (2)	C6—C7	1.387 (3)
N2—C6	1.336 (2)	C6—H6	0.9500
N2—C10	1.347 (2)	C7—C8	1.385 (3)
O1—C3	1.434 (2)	C7—H7	0.9500
O1—H1O	0.832 (10)	C8—C9	1.386 (3)
O2—C5	1.422 (2)	C8—H8	0.9500
O2—H2O	0.830 (10)	C9—C10	1.393 (2)
O1W—H1W	0.835 (10)	C9—H9	0.9500
O1W—H2W	0.832 (9)	C10—C10^i^	1.485 (3)
			
N2^i^—Zn—N2	75.71 (8)	H2A—C2—H2B	107.6
N2^i^—Zn—S2^i^	92.61 (4)	O1—C3—C2	109.67 (15)
N2—Zn—S2^i^	102.13 (4)	O1—C3—H3A	109.7
N2^i^—Zn—S2	102.13 (4)	C2—C3—H3A	109.7
N2—Zn—S2	92.61 (4)	O1—C3—H3B	109.7
S2^i^—Zn—S2	161.36 (2)	C2—C3—H3B	109.7
N2^i^—Zn—S1^i^	159.33 (4)	H3A—C3—H3B	108.2
N2—Zn—S1^i^	94.50 (4)	N1—C4—C5	113.40 (15)
S2^i^—Zn—S1^i^	71.377 (15)	N1—C4—H4A	108.9
S2—Zn—S1^i^	96.394 (15)	C5—C4—H4A	108.9
N2^i^—Zn—S1	94.50 (4)	N1—C4—H4B	108.9
N2—Zn—S1	159.33 (4)	C5—C4—H4B	108.9
S2^i^—Zn—S1	96.394 (15)	H4A—C4—H4B	107.7
S2—Zn—S1	71.376 (15)	O2—C5—C4	114.03 (16)
S1^i^—Zn—S1	100.09 (2)	O2—C5—H5A	108.7
C1—S1—Zn	85.11 (6)	C4—C5—H5A	108.7
C1—S2—Zn	85.62 (6)	O2—C5—H5B	108.7
C1—N1—C2	120.14 (14)	C4—C5—H5B	108.7
C1—N1—C4	120.76 (15)	H5A—C5—H5B	107.6
C2—N1—C4	119.02 (14)	N2—C6—C7	122.73 (17)
C6—N2—C10	118.74 (15)	N2—C6—H6	118.6
C6—N2—Zn	124.56 (12)	C7—C6—H6	118.6
C10—N2—Zn	115.97 (11)	C8—C7—C6	118.61 (17)
C3—O1—H1O	107.5 (17)	C8—C7—H7	120.7
C5—O2—H2O	109.3 (18)	C6—C7—H7	120.7
H1W—O1W—H2W	105 (2)	C7—C8—C9	119.16 (17)
N1—C1—S1	121.46 (13)	C7—C8—H8	120.4
N1—C1—S2	120.88 (13)	C9—C8—H8	120.4
S1—C1—S2	117.64 (10)	C8—C9—C10	118.85 (16)
N1—C2—C3	114.22 (15)	C8—C9—H9	120.6
N1—C2—H2A	108.7	C10—C9—H9	120.6
C3—C2—H2A	108.7	N2—C10—C9	121.89 (16)
N1—C2—H2B	108.7	N2—C10—C10^i^	115.54 (10)
C3—C2—H2B	108.7	C9—C10—C10^i^	122.56 (11)
			
C2—N1—C1—S1	4.9 (2)	N1—C4—C5—O2	85.17 (19)
C4—N1—C1—S1	−178.34 (12)	C10—N2—C6—C7	1.4 (3)
C2—N1—C1—S2	−173.68 (12)	Zn—N2—C6—C7	−168.48 (14)
C4—N1—C1—S2	3.1 (2)	N2—C6—C7—C8	−0.1 (3)
Zn—S1—C1—N1	−173.88 (14)	C6—C7—C8—C9	−1.1 (3)
Zn—S1—C1—S2	4.70 (9)	C7—C8—C9—C10	1.1 (3)
Zn—S2—C1—N1	173.85 (14)	C6—N2—C10—C9	−1.4 (3)
Zn—S2—C1—S1	−4.73 (9)	Zn—N2—C10—C9	169.27 (13)
C1—N1—C2—C3	−81.8 (2)	C6—N2—C10—C10^i^	179.44 (18)
C4—N1—C2—C3	101.31 (18)	Zn—N2—C10—C10^i^	−9.8 (2)
N1—C2—C3—O1	−80.47 (19)	C8—C9—C10—N2	0.2 (3)
C1—N1—C4—C5	86.7 (2)	C8—C9—C10—C10^i^	179.3 (2)
C2—N1—C4—C5	−96.50 (19)		

Symmetry code: (i) −*x*+3/2, −*y*+1/2, *z*.

### (I) (2,2'-Bipyridyl-κ2N,N')bis[N,N-bis(2-hydroxyethyl)dithiocarbamato-κ2S,S']zinc dihydrate Hydrogen-bond geometry (Å, º)

**Table:**

*D*—H···*A*	*D*—H	H···*A*	*D*···*A*	*D*—H···*A*
O2—H2*O*···O1	0.83 (2)	1.87 (2)	2.696 (2)	177 (3)
O1—H1*O*···O1*W*	0.83 (2)	1.88 (2)	2.7115 (19)	177 (2)
O1*W*—H1*W*···O2^ii^	0.83 (2)	1.91 (2)	2.7216 (19)	166 (2)
O1*W*—H2*W*···S2^iii^	0.83 (2)	2.45 (2)	3.2733 (15)	170 (2)
C7—H7···O1*W*^iv^	0.95	2.58	3.517 (2)	171
C6—H6···S2^v^	0.95	2.81	3.490 (2)	129
C9—H9···S1^vi^	0.95	2.84	3.6857 (18)	149

Symmetry codes: (ii) *x*+1, *y*, *z*; (iii) *x*+1/2, −*y*+1, −*z*+1/2; (iv) −*x*+1, *y*−1/2, −*z*+1/2; (v) −*x*+1/2, −*y*+1/2, *z*; (vi) −*x*+3/2, *y*, *z*+1/2.

### (II) (2,2'-Bipyridyl-κ2N,N')bis[N-(2-hydroxyethyl)-N-isopropyldithiocarbamato-κ2S,S']zinc Crystal data

**Table:**

[Zn(C_6_H_12_NOS_2_)_2_(C_10_H_8_N_2_)]	*F*(000) = 1208
*M**_r_* = 578.12	*D*_x_ = 1.422 Mg m^−^^3^
Monoclinic, *C*2/*c*	Mo *K*α radiation, λ = 0.71073 Å
*a* = 19.4997 (11) Å	Cell parameters from 3771 reflections
*b* = 9.0027 (5) Å	θ = 2.3–27.5°
*c* = 15.5352 (8) Å	µ = 1.25 mm^−^^1^
β = 98.031 (5)°	*T* = 100 K
*V* = 2700.5 (3) Å^3^	Prism, light-yellow
*Z* = 4	0.25 × 0.25 × 0.15 mm

### (II) (2,2'-Bipyridyl-κ2N,N')bis[N-(2-hydroxyethyl)-N-isopropyldithiocarbamato-κ2S,S']zinc Data collection

**Table:**

Agilent SuperNova Dual diffractometer with Atlas detector	3095 independent reflections
Radiation source: SuperNova (Mo) X-ray Source	2657 reflections with *I* > 2σ(*I*)
Mirror monochromator	*R*_int_ = 0.048
Detector resolution: 10.4041 pixels mm^-1^	θ_max_ = 27.5°, θ_min_ = 2.5°
ω scan	*h* = −21→25
Absorption correction: multi-scan (*CrysAlis PRO*; Agilent, 2012)	*k* = −11→10
*T*_min_ = 0.737, *T*_max_ = 1.000	*l* = −20→20
11190 measured reflections	

### (II) (2,2'-Bipyridyl-κ2N,N')bis[N-(2-hydroxyethyl)-N-isopropyldithiocarbamato-κ2S,S']zinc Refinement

**Table:**

Refinement on *F*^2^	1 restraint
Least-squares matrix: full	Hydrogen site location: mixed
*R*[*F*^2^ > 2σ(*F*^2^)] = 0.030	*w* = 1/[σ^2^(*F*_o_^2^) + (0.0309*P*)^2^ + 1.2812*P*] where *P* = (*F*_o_^2^ + 2*F*_c_^2^)/3
*wR*(*F*^2^) = 0.073	(Δ/σ)_max_ = 0.001
*S* = 1.03	Δρ_max_ = 0.38 e Å^−^^3^
3095 reflections	Δρ_min_ = −0.35 e Å^−^^3^
155 parameters	

### (II) (2,2'-Bipyridyl-κ2N,N')bis[N-(2-hydroxyethyl)-N-isopropyldithiocarbamato-κ2S,S']zinc Special details

**Table:**

Geometry. All esds (except the esd in the dihedral angle between two l.s. planes) are estimated using the full covariance matrix. The cell esds are taken into account individually in the estimation of esds in distances, angles and torsion angles; correlations between esds in cell parameters are only used when they are defined by crystal symmetry. An approximate (isotropic) treatment of cell esds is used for estimating esds involving l.s. planes.

### (II) (2,2'-Bipyridyl-κ2N,N')bis[N-(2-hydroxyethyl)-N-isopropyldithiocarbamato-κ2S,S']zinc Fractional atomic coordinates and isotropic or equivalent isotropic displacement parameters (Å^2^)

**Table:**

	*x*	*y*	*z*	*U*_iso_*/*U*_eq_	
Zn	0.5000	0.74113 (3)	0.7500	0.01278 (9)	
S1	0.58946 (2)	0.56650 (5)	0.82428 (3)	0.01649 (12)	
S2	0.58906 (2)	0.69208 (5)	0.65006 (3)	0.01671 (12)	
O1	0.81368 (8)	0.48579 (17)	0.82921 (10)	0.0311 (4)	
H1O	0.8403 (11)	0.416 (2)	0.8210 (17)	0.047*	
N1	0.67544 (8)	0.47424 (17)	0.71467 (10)	0.0146 (3)	
N2	0.46566 (8)	0.93220 (16)	0.67036 (10)	0.0138 (3)	
C1	0.62467 (10)	0.56746 (19)	0.72861 (12)	0.0141 (4)	
C2	0.70163 (10)	0.3630 (2)	0.78132 (12)	0.0193 (4)	
H2A	0.7249	0.2822	0.7533	0.023*	
H2B	0.6619	0.3191	0.8056	0.023*	
C3	0.75234 (11)	0.4277 (2)	0.85548 (13)	0.0270 (5)	
H3A	0.7288	0.5078	0.8837	0.032*	
H3B	0.7651	0.3491	0.8994	0.032*	
C4	0.69560 (10)	0.4583 (2)	0.62598 (12)	0.0182 (4)	
H4	0.6837	0.5539	0.5946	0.022*	
C5	0.65197 (11)	0.3372 (2)	0.57632 (13)	0.0256 (5)	
H5A	0.6028	0.3617	0.5737	0.038*	
H5B	0.6613	0.2419	0.6061	0.038*	
H5C	0.6638	0.3301	0.5172	0.038*	
C6	0.77272 (11)	0.4326 (3)	0.62649 (14)	0.0273 (5)	
H6A	0.7989	0.5095	0.6617	0.041*	
H6B	0.7836	0.4371	0.5668	0.041*	
H6C	0.7854	0.3347	0.6513	0.041*	
C7	0.43644 (10)	0.9239 (2)	0.58712 (12)	0.0169 (4)	
H7	0.4245	0.8288	0.5631	0.020*	
C8	0.42296 (10)	1.0474 (2)	0.53475 (12)	0.0208 (4)	
H8	0.4014	1.0375	0.4763	0.025*	
C9	0.44151 (11)	1.1856 (2)	0.56917 (13)	0.0242 (5)	
H9	0.4330	1.2725	0.5346	0.029*	
C10	0.47270 (11)	1.1956 (2)	0.65477 (13)	0.0212 (4)	
H10	0.4863	1.2894	0.6795	0.025*	
C11	0.48384 (10)	1.0669 (2)	0.70400 (11)	0.0151 (4)	

### (II) (2,2'-Bipyridyl-κ2N,N')bis[N-(2-hydroxyethyl)-N-isopropyldithiocarbamato-κ2S,S']zinc Atomic displacement parameters (Å^2^)

**Table:**

	*U*^11^	*U*^22^	*U*^33^	*U*^12^	*U*^13^	*U*^23^
Zn	0.01357 (17)	0.01108 (16)	0.01328 (16)	0.000	0.00045 (12)	0.000
S1	0.0208 (3)	0.0152 (2)	0.0139 (2)	0.00390 (18)	0.00368 (19)	0.00164 (17)
S2	0.0166 (3)	0.0176 (3)	0.0158 (2)	0.00287 (19)	0.00185 (18)	0.00486 (18)
O1	0.0267 (9)	0.0260 (9)	0.0369 (9)	0.0060 (7)	−0.0081 (7)	−0.0051 (7)
N1	0.0148 (8)	0.0147 (8)	0.0139 (8)	0.0021 (6)	0.0006 (6)	−0.0012 (6)
N2	0.0136 (8)	0.0141 (8)	0.0135 (8)	−0.0003 (6)	0.0015 (6)	−0.0003 (6)
C1	0.0145 (10)	0.0128 (9)	0.0142 (9)	−0.0027 (7)	−0.0008 (7)	−0.0016 (7)
C2	0.0231 (11)	0.0150 (10)	0.0191 (10)	0.0072 (8)	0.0004 (8)	0.0017 (8)
C3	0.0301 (13)	0.0275 (12)	0.0208 (11)	0.0131 (9)	−0.0054 (9)	−0.0023 (9)
C4	0.0202 (10)	0.0195 (10)	0.0157 (10)	0.0018 (8)	0.0053 (8)	−0.0018 (7)
C5	0.0280 (12)	0.0292 (12)	0.0191 (10)	−0.0040 (9)	0.0020 (9)	−0.0073 (9)
C6	0.0200 (11)	0.0360 (13)	0.0266 (12)	0.0052 (9)	0.0051 (9)	−0.0051 (9)
C7	0.0150 (10)	0.0201 (10)	0.0152 (9)	−0.0009 (7)	0.0006 (7)	−0.0020 (7)
C8	0.0199 (11)	0.0300 (12)	0.0119 (9)	0.0049 (8)	0.0002 (8)	0.0041 (8)
C9	0.0296 (12)	0.0224 (11)	0.0211 (11)	0.0079 (9)	0.0058 (9)	0.0099 (8)
C10	0.0304 (12)	0.0127 (10)	0.0205 (10)	0.0032 (8)	0.0042 (9)	0.0015 (8)
C11	0.0166 (10)	0.0144 (9)	0.0149 (10)	0.0022 (7)	0.0041 (8)	−0.0007 (7)

### (II) (2,2'-Bipyridyl-κ2N,N')bis[N-(2-hydroxyethyl)-N-isopropyldithiocarbamato-κ2S,S']zinc Geometric parameters (Å, º)

**Table:**

Zn—N2^i^	2.1695 (15)	C3—H3B	0.9900
Zn—N2	2.1695 (15)	C4—C6	1.521 (3)
Zn—S1	2.5068 (5)	C4—C5	1.525 (3)
Zn—S1^i^	2.5068 (5)	C4—H4	1.0000
Zn—S2^i^	2.5247 (5)	C5—H5A	0.9800
Zn—S2	2.5247 (5)	C5—H5B	0.9800
S1—C1	1.7221 (19)	C5—H5C	0.9800
S2—C1	1.7301 (18)	C6—H6A	0.9800
O1—C3	1.417 (3)	C6—H6B	0.9800
O1—H1O	0.833 (10)	C6—H6C	0.9800
N1—C1	1.338 (2)	C7—C8	1.381 (3)
N1—C2	1.479 (2)	C7—H7	0.9500
N1—C4	1.492 (2)	C8—C9	1.382 (3)
N2—C7	1.340 (2)	C8—H8	0.9500
N2—C11	1.348 (2)	C9—C10	1.386 (3)
C2—C3	1.525 (3)	C9—H9	0.9500
C2—H2A	0.9900	C10—C11	1.388 (3)
C2—H2B	0.9900	C10—H10	0.9500
C3—H3A	0.9900	C11—C11^i^	1.479 (4)
			
N2^i^—Zn—N2	75.08 (8)	O1—C3—H3B	108.7
N2^i^—Zn—S1	95.47 (4)	C2—C3—H3B	108.7
N2—Zn—S1	154.06 (4)	H3A—C3—H3B	107.6
N2^i^—Zn—S1^i^	154.07 (4)	N1—C4—C6	113.45 (16)
N2—Zn—S1^i^	95.47 (4)	N1—C4—C5	109.62 (16)
S1—Zn—S1^i^	102.32 (2)	C6—C4—C5	112.05 (17)
N2^i^—Zn—S2^i^	88.40 (4)	N1—C4—H4	107.1
N2—Zn—S2^i^	107.78 (4)	C6—C4—H4	107.1
S1—Zn—S2^i^	95.822 (17)	C5—C4—H4	107.1
S1^i^—Zn—S2^i^	71.289 (16)	C4—C5—H5A	109.5
N2^i^—Zn—S2	107.78 (4)	C4—C5—H5B	109.5
N2—Zn—S2	88.39 (4)	H5A—C5—H5B	109.5
S1—Zn—S2	71.288 (16)	C4—C5—H5C	109.5
S1^i^—Zn—S2	95.822 (17)	H5A—C5—H5C	109.5
S2^i^—Zn—S2	159.86 (3)	H5B—C5—H5C	109.5
C1—S1—Zn	86.29 (6)	C4—C6—H6A	109.5
C1—S2—Zn	85.55 (6)	C4—C6—H6B	109.5
C3—O1—H1O	109.6 (19)	H6A—C6—H6B	109.5
C1—N1—C2	120.14 (15)	C4—C6—H6C	109.5
C1—N1—C4	120.40 (15)	H6A—C6—H6C	109.5
C2—N1—C4	118.15 (14)	H6B—C6—H6C	109.5
C7—N2—C11	118.51 (16)	N2—C7—C8	122.95 (17)
C7—N2—Zn	124.18 (12)	N2—C7—H7	118.5
C11—N2—Zn	116.66 (12)	C8—C7—H7	118.5
N1—C1—S1	121.98 (14)	C7—C8—C9	118.59 (18)
N1—C1—S2	121.71 (14)	C7—C8—H8	120.7
S1—C1—S2	116.28 (11)	C9—C8—H8	120.7
N1—C2—C3	113.23 (16)	C8—C9—C10	119.06 (18)
N1—C2—H2A	108.9	C8—C9—H9	120.5
C3—C2—H2A	108.9	C10—C9—H9	120.5
N1—C2—H2B	108.9	C9—C10—C11	119.24 (18)
C3—C2—H2B	108.9	C9—C10—H10	120.4
H2A—C2—H2B	107.7	C11—C10—H10	120.4
O1—C3—C2	114.06 (17)	N2—C11—C10	121.63 (17)
O1—C3—H3A	108.7	N2—C11—C11^i^	115.33 (10)
C2—C3—H3A	108.7	C10—C11—C11^i^	123.03 (11)
			
C2—N1—C1—S1	1.8 (2)	C1—N1—C4—C5	−89.1 (2)
C4—N1—C1—S1	168.55 (13)	C2—N1—C4—C5	77.9 (2)
C2—N1—C1—S2	−176.25 (13)	C11—N2—C7—C8	−1.2 (3)
C4—N1—C1—S2	−9.5 (2)	Zn—N2—C7—C8	−171.67 (14)
Zn—S1—C1—N1	−170.98 (15)	N2—C7—C8—C9	1.2 (3)
Zn—S1—C1—S2	7.21 (9)	C7—C8—C9—C10	−0.2 (3)
Zn—S2—C1—N1	171.03 (15)	C8—C9—C10—C11	−0.7 (3)
Zn—S2—C1—S1	−7.16 (9)	C7—N2—C11—C10	0.3 (3)
C1—N1—C2—C3	−79.6 (2)	Zn—N2—C11—C10	171.46 (14)
C4—N1—C2—C3	113.39 (18)	C7—N2—C11—C11^i^	−179.62 (19)
N1—C2—C3—O1	−62.3 (2)	Zn—N2—C11—C11^i^	−8.5 (3)
C1—N1—C4—C6	144.80 (18)	C9—C10—C11—N2	0.7 (3)
C2—N1—C4—C6	−48.2 (2)	C9—C10—C11—C11^i^	−179.4 (2)

Symmetry code: (i) −*x*+1, *y*, −*z*+3/2.

### (II) (2,2'-Bipyridyl-κ2N,N')bis[N-(2-hydroxyethyl)-N-isopropyldithiocarbamato-κ2S,S']zinc Hydrogen-bond geometry (Å, º)

**Table:**

*D*—H···*A*	*D*—H	H···*A*	*D*···*A*	*D*—H···*A*
O1—H1*O*···S2^ii^	0.84 (2)	2.45 (2)	3.2437 (16)	160 (2)
C5—H5*B*···O1^ii^	0.98	2.54	3.512 (2)	175
C9—H9···S2^iii^	0.95	2.86	3.550 (2)	130

Symmetry codes: (ii) −*x*+3/2, *y*−1/2, −*z*+3/2; (iii) −*x*+1, −*y*+2, −*z*+1.
